# Is there anybody in there? Entomological evidence from a boat burial at Øksnes in Vesterålen, northern Norway

**DOI:** 10.1371/journal.pone.0200545

**Published:** 2018-07-27

**Authors:** Eva Panagiotakopulu, Paul C. Buckland, Stephen Wickler

**Affiliations:** 1 School of Geosciences, University of Edinburgh, Edinburgh, United Kingdom; 2 Den Bank Close, Sheffield, United Kingdom; 3 Tromsø University Museum, Department of Cultural Sciences, University of Tromsø, Tromsø, Norway; New York State Museum, UNITED STATES

## Abstract

Although there are several well preserved Viking boat burials from Norway, until recently palaeoecological research on their context has often been limited. Research on fossil insect remains in particular can provide valuable forensic information even in the absence of an actual body. Here we present archaeoentomological information from a boat burial at Øksnes in Vesterålen, northeast Norway, an area where Norse and Sami traditions overlap. Excavated in 1934, organic preservation from the burial was limited to parts of the boat and a clump of bird feathers which were preserved in the Tromsø University Museum, and from which fossil insects were recovered. The insect assemblage from Øksnes includes the blowfly, *Protophormia terraenovae* (Rob.-Des.), which indicates exposure of the body and the probable timing of the burial. The high numbers of the human flea, *Pulex irritans* L. from among the feathers, suggests that these, probably from a pillow under the corpse, originated from within a domestic context. Deposition of flowers as part of the burial is discussed on the basis of the insect fauna. The absence of a body and any associated post burial decay fauna implies its exhumation and disposal elsewhere and this is discussed in the context of other exhumed medieval burials and Saga and other sources.

## Introduction

‘Bog bodies,’ burials, usually solitary, preserved in wetlands, have been the source of endless fascination amongst both the general public and the more specialist archaeological community [1–4]. Examples, usually found during the process of peat cutting [5–6], range in date from the Late Bronze Age to the medieval period and inferred reasons for burial include murder victims, sacrifice to chthonic deities, disposal of potential zombies or simply those lost on the moors. Classical, Saga and later sources provide examples of all. To this list must be added more formal burials in containers, either coffins or boats, where at least partial preservation has been engendered by either anaerobic conditions or waterlogging, the Viking age ship burials of Gokstad and Oseberg being perhaps the best known examples [7]. Similarly furnished interments are widespread around the North Sea and Baltic at least from the seventh century to the imposition of Christianity, with recently excavated examples ranging from Scar on Sanday, Orkney [8] and Swordle Bay on Ardnamurchan, western Scotland [9] to Salma in Estonia [10]. Older finds, however, may still provide new evidence where material survives in museum collections and this paper considers a find made in Arctic Norway nearly a century ago and preserved in the Tromsø University Museum. It uses the entomological evidence to examine the nature and context of the grave and the absence of a body.

## The context

**Fig 1:**
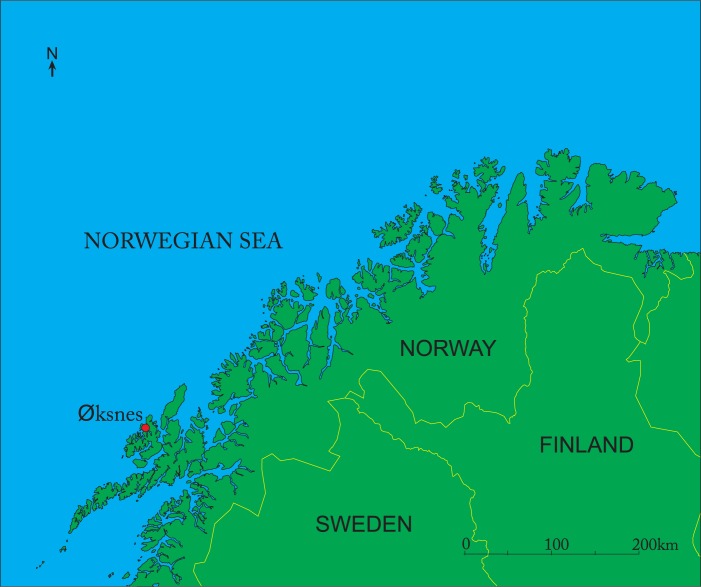
Location map of Øksnes, northern Norway.

**Fig 2:**
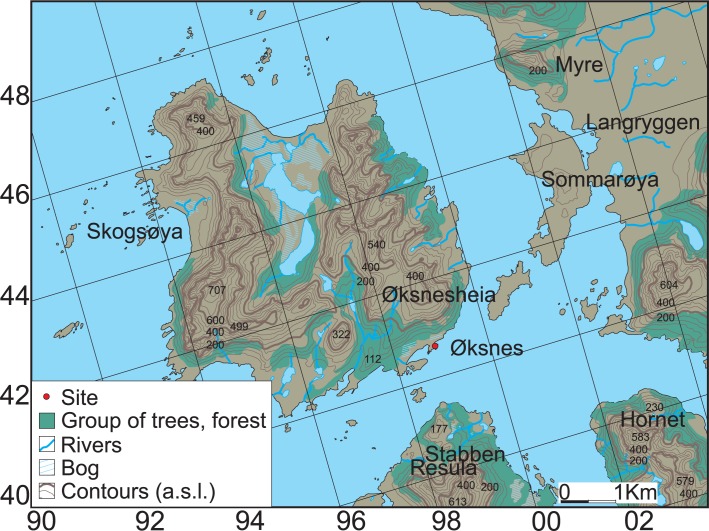
Topographic map of Øksnes on Skogsøya in Vesterålen islands.

**Fig 3:**
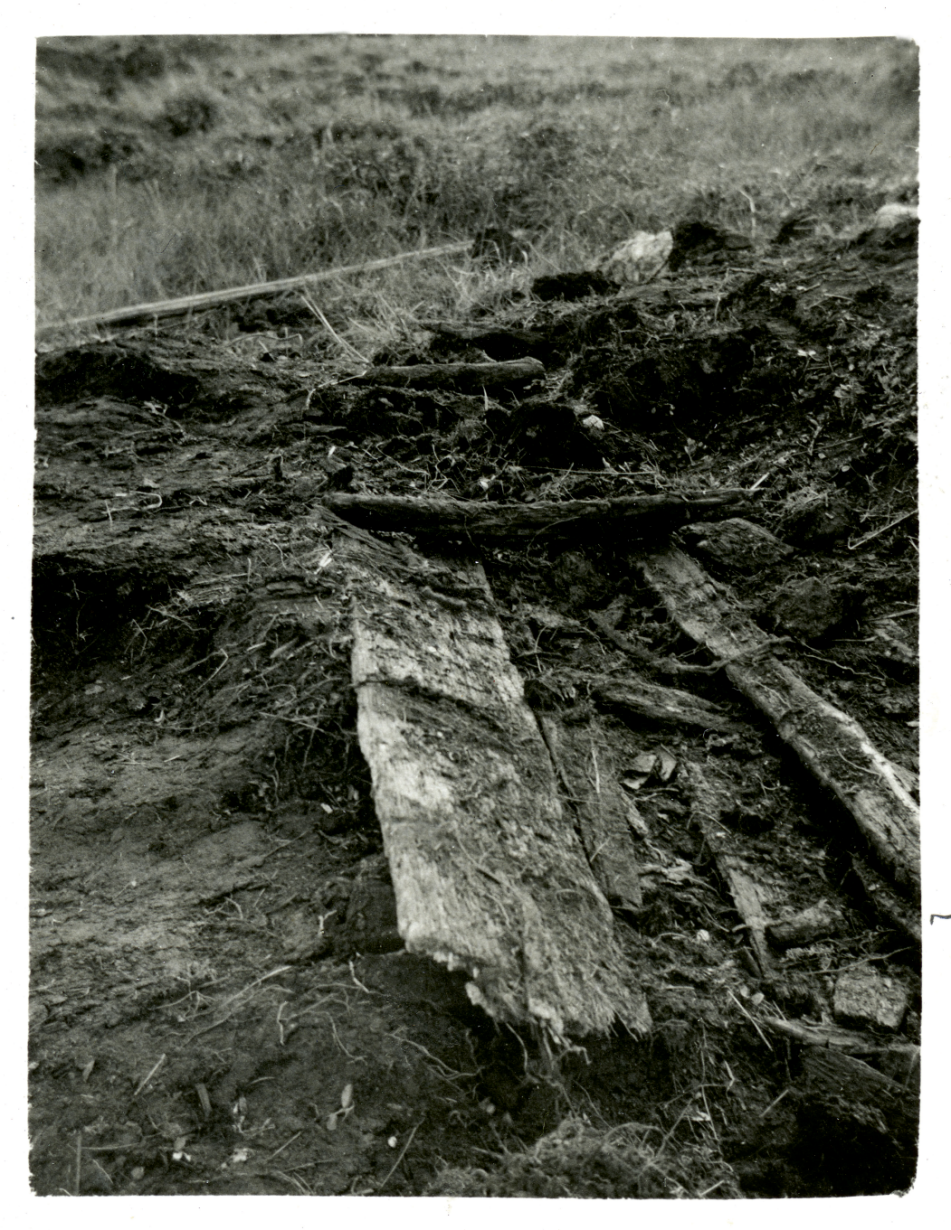
Photograph of the Øksnes boat burial excavation from Gjessing 1941.

During road construction in 1934 at Øksnes on Skogsøya in Vesterålen, an island group off the northwest coast of Norway, Lat. 68° 52’ 38”N; Long. 14° 58’ 22”E (Figs 1 and 2), the base of a wooden boat estimated to have been 8-10m long, was recovered from an adjacent peatbog (Fig 3).

**Fig 4:**
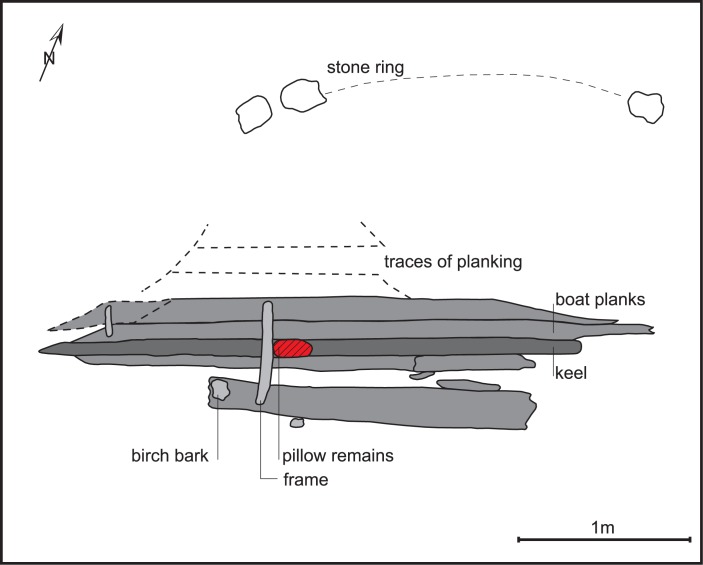
Plan drawing of the Øksnes boat burial redrawn from Gjessing 1941 by Adnan Icagic, Tromsø University Museum.

In the publication of the excavation results, Gjessing [11] argued that the boat was part of a burial from the Viking Age (AD 800–1050). Beneath a low mound in the bog, the boat lay within a stone ring or kerbing (Fig 4). Unfortunately, earlier peat cutting had led to the loss of the bow and stern sections of the boat, and less than 4m of the midsection survived, including the keel, with two planks either side and the outline of two additional planks with caulking remains on one side, and two frame fragments (Fig 2). The planks had been sewn together with a discontinuous technique using twined root fibres passing through paired sets of holes along the plank edges. The planking joints had been caulked with spun wool twisted into narrow strips. Gjessing (idem) suggests that the boat had been covered with birch bark as remains of this were recovered under the boat planks.

Details of the position of any burial within the boat are uncertain and the only surviving grave goods were a mass of feathers and fragments of woollen textile, interpreted as a pillow or duvet by Gjessing (idem), an iron axe and hair from a hide. The pillow may have placed beneath the head of a corpse (Fig 3), but there was no evidence of a body. Despite the heavily corroded state of the axe, Gjessing was able to relate it to a typology of similar pieces from Viking contexts (Jan Petersen Type E) dated from the second half of the 9th century to early 10th century. The coarse woollen cloth had been woven using a technique which was quite common in the Viking period [12], and the age has been confirmed by radiocarbon dating to 888–994 cal AD at two sigma (TRa-2953). The feathers have been identified as belonging predominantly to ‘white headed’ gulls, Lariidae, and part of the assemblage was identified to belong to ducks, Anseriformes, possibly eider, and to the cormorant *Phalacrocorax carbo* (L.) [13]. Clusters of animal hair were also found with the textile and Kirkinen [14] suggests that these had been attached to a hide which had been used to wrap the body of the deceased, although it could equally have been part of a cloak or other clothing. Kirkinen (ibid) has identified the hair as deriving from a bovid, most likely representing domestic cattle (*Bos taurus* L.).

Although the Øksnes find and its deposition is unique in terms of detail, it resembles other boat burials from northern Norway, including a boat grave from Føre, also in Vesterålen not far from Øksnes, excavated in 1989 [15]. This boat, however, was clinker-built with iron rivets and contained the remains of a female with a variety of grave goods, including an axe, a more typical Viking Age burial. Of the more than 30 Iron Age boat burials documented in northern Norway, predominantly from the Viking Age, the Øksnes find is the only instance of interment in a bog.

## Methodology

**Fig 5:**
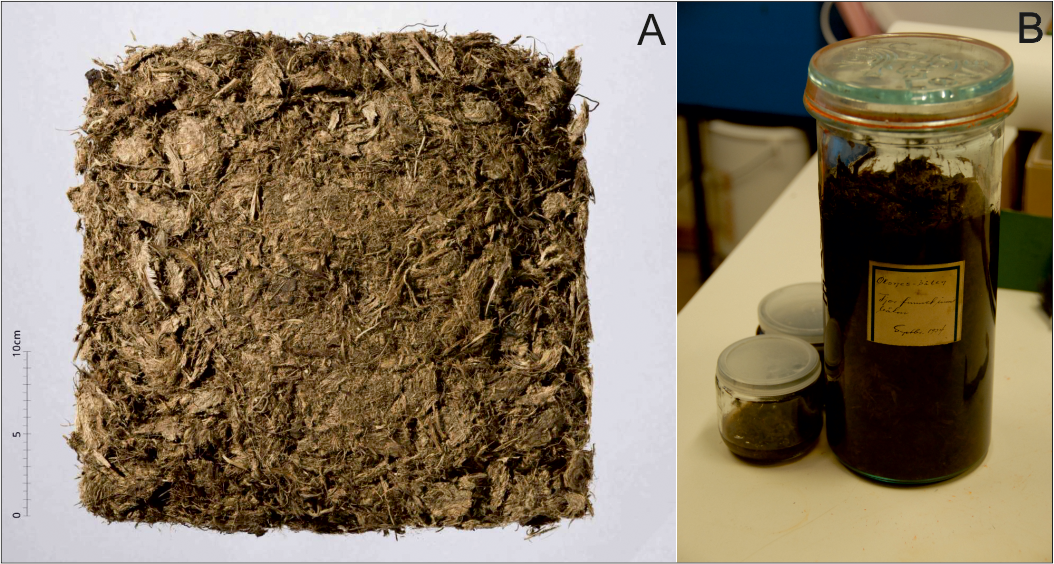
Photograph of the Øksnes feathers A. kept as found B. preserved in an ethanol based liquid.

The feathers from the Øksnes burial are preserved in the Tromsø University Museum and were made available for study (Fig 5). The greater part of the feathers is kept as recovered, while a sample of the material had been placed in an ethanol based liquid shortly after excavation. Both of these were subsampled and studied under a low power stereomicroscope. On close inspection, it was decided that the material which was kept as recovered (Fig 5), without use of chemicals, was more appropriate for this study as the ethanol based medium had become dark over time and was strongly aromatic, which made microscope study difficult. About 1/6 of the material was examined during two visits to Tromsø and the insect remains separated. These were identified using the Osborne Collection of Coleoptera, housed in the School of Geosciences, Edinburgh University, and relevant entomological keys. Although during handling the material tended to be fragile and prone to further fragmentation, preservation was very good and identification to species level was possible in most cases. The insects are currently deposited at the School of GeoSciences, University of Edinburgh.

## Results

The material sorted from the feathers includes beetles (Coleoptera), flies (Diptera) and fleas (Siphonaptera). The results are presented in Table 1, where Coleopteran taxonomy follows Böhme [16].

**Table 1:** Insect assemblage from the Øksnes burial.

Taxa	S1
Coleoptera	
Carabidae	
*Patrobus atrorufus* (Strom.)	1^a^
Hydrophilidae	
*Cercyon haemorrhoidalis* (F.)	1
*Cryptopleurum minutum* (Fab.)	1
Leiodidae	
*Catops* sp.	1
Staphylinidae	
*Phyllodrepa floralis* (Payk.)	1
*Anthobium melanocephalum* (Ill.)	1
*Olophrum assimile* (Payk.)	1
*Othius* sp.	1
Elateridae	
*Athous subfuscus* (Müll.) (larva)	1
Byturidae	
*Byturus tomentosus* (Deg.)	1
Latriidae	
*Latridius pseudominutus* (Strand)	1
Siphonaptera	
Pulicidae	
*Pulex irritans* L.	35
Diptera	
Heleomyzidae	1
*Heleomyza borealis* Bohe.	1
Sphaeroceridae indet.	1
Calliphoridae	
*Protophormia terraenovae* (Rob.-Des.)	12

^a^The numbers represent Minimum Numbers of Individuals (MNIs).

**Fig 6:**
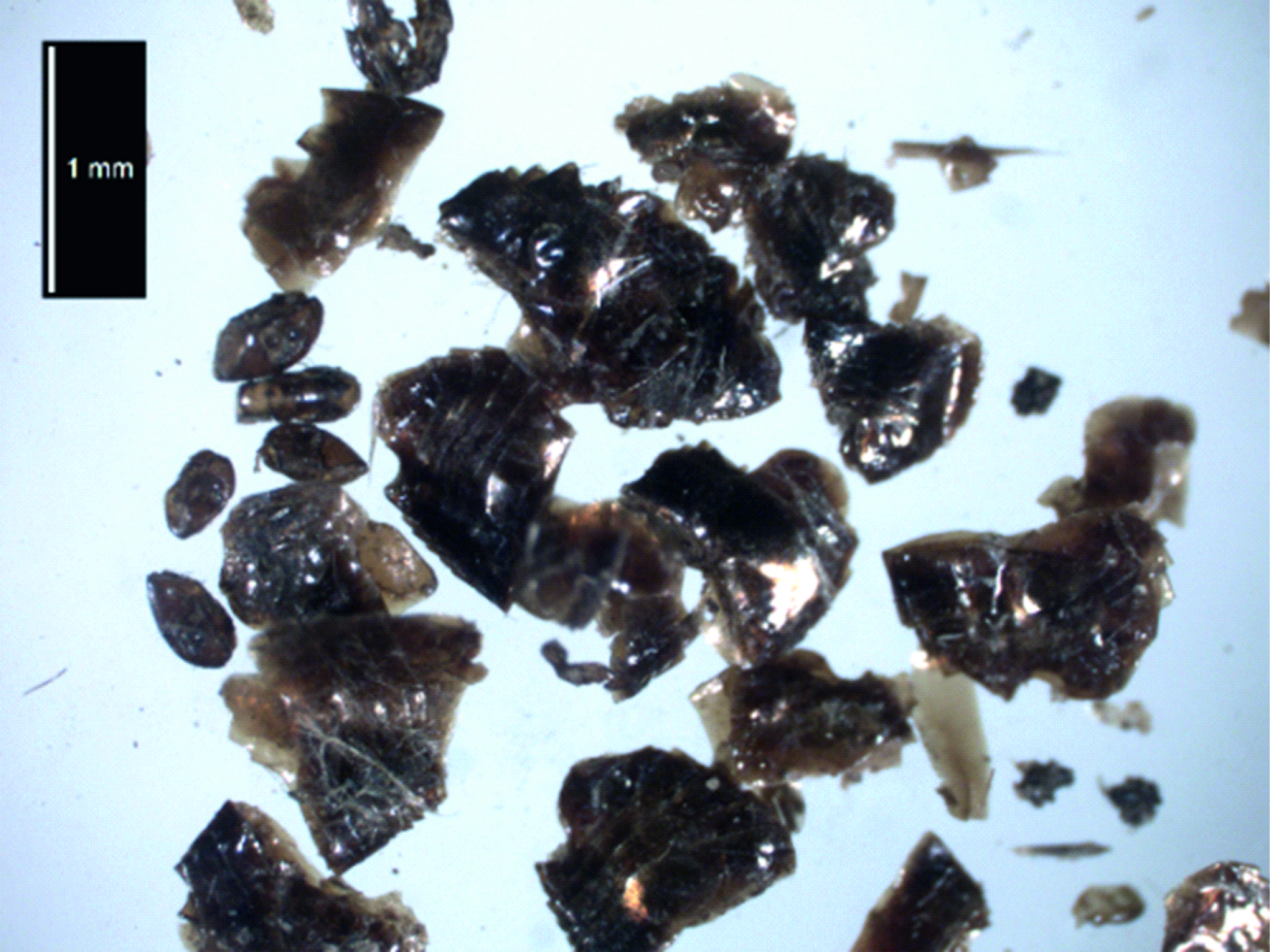
Fragmented specimens of human fleas, *Pulex irritans* L., recovered from amongst the pillow feathers.

Thirty-five specimens of the human flea, *Pulex irritans* L., were recovered from amongst the feathers (Fig 6). *P*. *irritans* is now a cosmopolitan, if increasingly rare ectoparasite on humans. It is also recorded from domestic animals, cat, dog and pig, and also from badger and fox, with casual occurrence on a wide range of other hosts [17]. Biogeographic and phylogenetic research place the origins of this species in South America with the Guinea pig as a primary host [18–19]. Although known for their ability to jump and reach alternative hosts, breeding requires animals with a relatively permanent abode, a base camp, home or nest [20]. In low temperatures, human fleas can survive for several months in the clothes of their hosts, and within domestic areas, including barns and stables, where body heat and decaying of excreta of potential hosts provides an artificially warmed habitat. Flea eggs may hatch into larvae in about 3–4 days, or longer depending on microclimate. The flea larvae, are eyeless and avoid light [21], feeding primarily on adult flea debris, dried faecal blood from adult fleas and infertile flea eggs [22–24]. They have been reported to consume other organic materials, including skin and feathers, and the pillow would initially have provided habitat for them. Temperature and humidity are, however, important for the survival of the larvae [25, 26], and they would not be able to withstand prolonged temperatures below freezing. Larvae generally undergo three moults and after a pupal stage of roughly four weeks spent in a cocoon constructed of faecal and other debris, the adults emerge and search for their first blood meal [25]. Human flea infestations may be severe [27] and they may also be secondary vectors of Plague. As would be expected, the European fossil record of *P*. *irritans* is extensive, ranging from the Neolithic in the south of France to medieval Oslo [28]. Its most northerly fossil record is from the clothing of sailors wrecked on Nova Zemlya in Barent’s ill-fated expedition of 1596–7 [29]. Improved hygiene, particularly the invention of the vacuum cleaner, has virtually eradicated the species from northern Europe and it was last recorded in Norway in 1948 [21].

**Fig 7:**
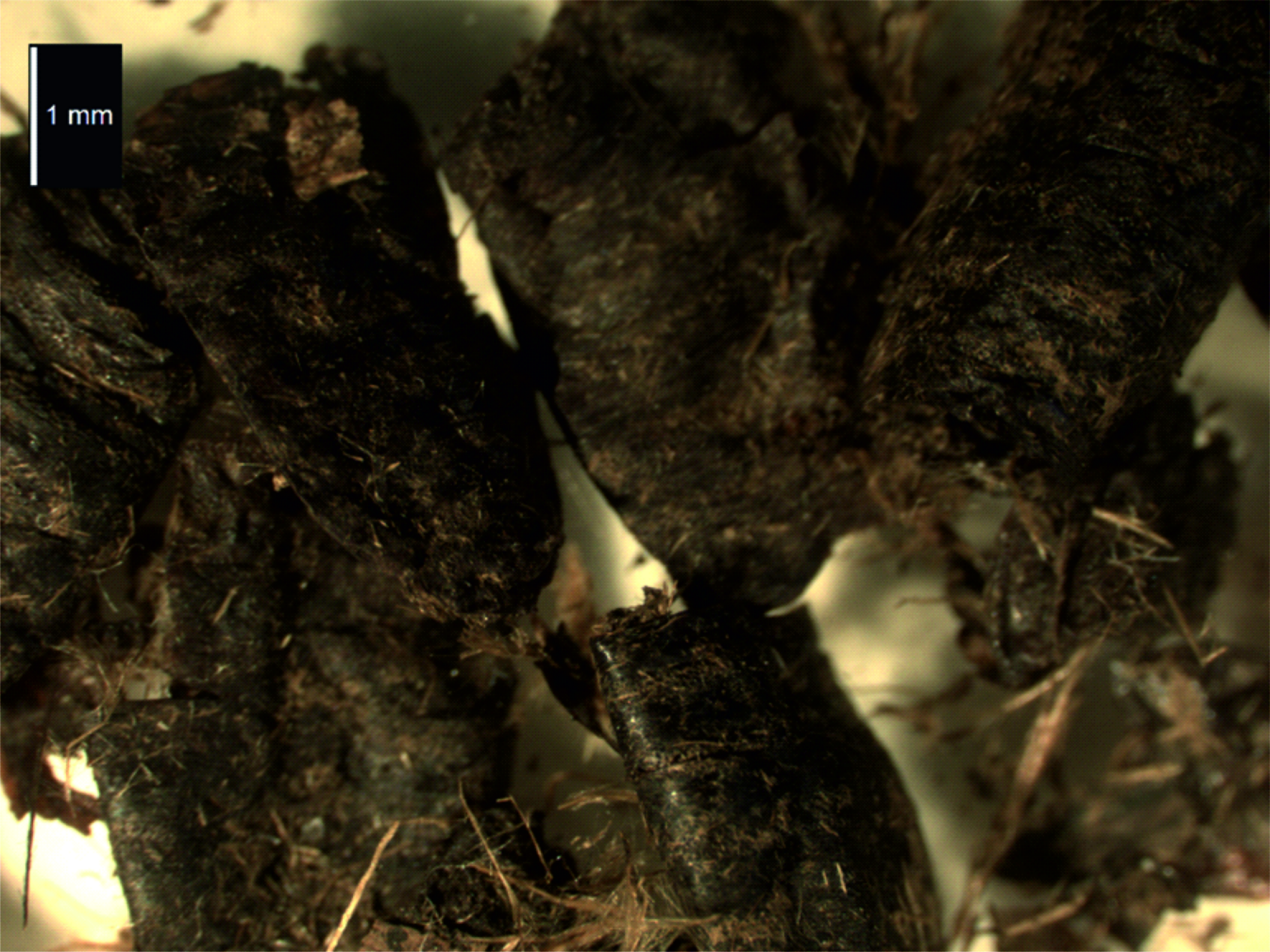
Calliphorid puparia, *Protophormia terraenovae* Bohe., recovered from the feathers in the Øksnes burial. Probably this was the area where the head of the dead person lay on the pillow and the maggots would have been feeding on the body.

The remainder of the insect assemblage is dominated by dipterous puparia, primarily blow flies, Calliphoridae (Fig 7). *Protophormia terraenovae* (Rob.-Des.) is widespread in the northern Holarctic and is common throughout Scandinavia [30]. Recorded frequently from garbage heaps as well as human and animal corpses, and occasionally responsible for myiasis in domestic animals, the fly oviposits on exposed rather than buried carrion [31]; Rognes [30] has noted a preference for human bodies in Finland, although this is clearly not exclusive [32–34]. The maggots tend to pupate on the carcasses [35–36], seldom moving more than 0.5m away from the corpse. Development takes around 11 days from egg to adult at 27°C, although the cycle might be protracted up to eight weeks in very low temperatures [35, 37]. Although cold resistant, it cannot breed in temperatures below 10°C and as a result in Scandinavia it tends to be more abundant in July. In Finland it is the most common blowfly in spring and it may have two generations in a year [30]. Erzinçlioğlu [38] noted the occurrence of its puparia in some numbers in the nasal cavities of one of the Lateglacial Condover mammoths from England and there are older records from similar places in woolly rhinoceros and steppe bison skulls from Belgium [39–41].

The fly assemblage also includes a puparium of *Heleomyza borealis* Bohe. and a fragmented sphaerocerid puparium. *H*. *borealis* feeds on proteins in decaying meat and faeces, preferring dark and squalid environments [42– 43]. It is cold resistant [44] and one of the few common flies in the High Arctic, with fossil records from Norse and Inuit sites in Greenland [45, 43]. Sphaerocerids are also often associated with forensic cases [31], although they occur widely in decaying plant and animal materials and many species have yet to be described in the larval stages [46]

**Fig 8:**
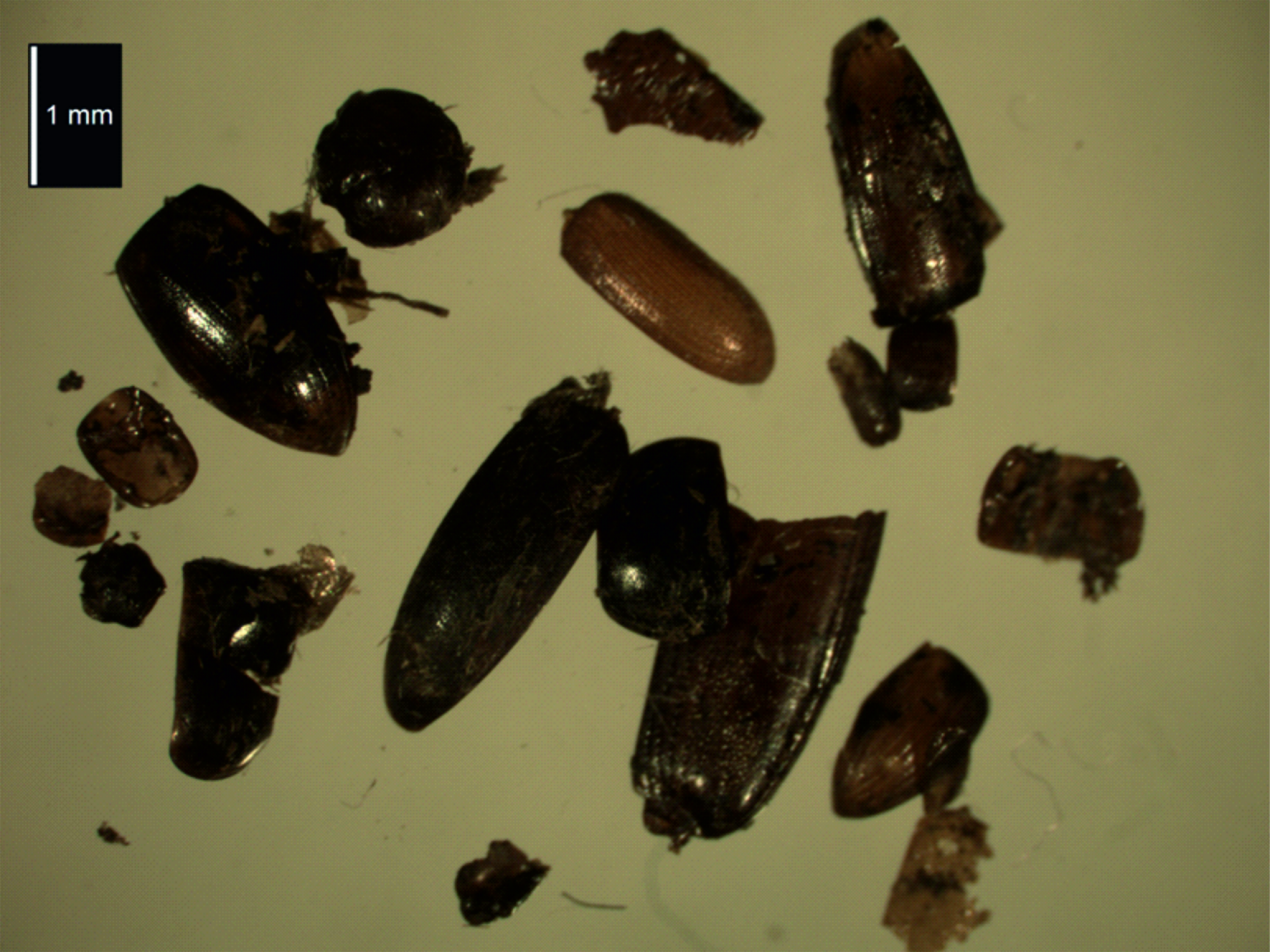
Beetles recovered from the feathers from the Øksnes burial.

The few beetle sclerites (Fig 8) were fragile and tended to break up during identification. Species are all represented by single individuals. The ground beetle *Patrobus atrorufus* (Strom.) is a eurytopic species, found in a variety of damp environments, from meadows to forests. It is favoured by human activities and may be found in pasture and cultivated areas [47]. *Cryptopleurum minutum* (Fab.) is similarly widespread, occurring in carrion as well as dung, compost and other decaying plant materials [48], habitats shared with the other hydrophilid present *Cercyon haemorroidalis* (F.) although this is also recorded from birds’ nests and in fresh carrion [49]; the genus *Catops* is also known from carrion, but can occur in plant litter [50]. As in most Arctic assemblages, there are examples of omaliine rove beetles. *Anthobium melanocephalum* (Ill.) is a species associated with forest litter but is also found in rotten fungi [51–52], while *Phyllodrepa floralis* (Payk.) is frequently synanthropic in the northern part of its range, breeding in hay and similar accumulations of plant debris [53–54]. *Olophrum assimile* (Payk.) is similarly a litter species, although it is not synanthropic. *Othius* sp. is found in leaf litter, heathland and woodland margins [49], Whilst the click beetle *Athous subfuscus* (Müll.) has been recorded from grassland and heather [55] and from woodland margins [49], its larvae live in the ground from November to May when they move closer to the upper soil layers to feed on pupae, larvae and cocoons of other insects [56].

All these species have Lateglacial and Holocene fossil records, although *A*. *melanocephalum* is only known from a single site in the Apennines [57].

The mould feeding beetle *Latridius assimilis* (Mann.) (= *pseudominutus* (Strand)) may be found in haystacks, in mouldy hay and straw, but also in fungi and occasionally under bark. In Scandinavia it has been collected in the wild in wood litter and mouldy vegetable matter, although much of the habitat data is confused with the similar *L*. *minutus* [58]; it appears to be largely synanthropic in the northern part of its range, occurring in cellars, barns, houses and stables; and it is a frequent component of archaeological insect assemblages including those from medieval Langenes in the Vesterålen islands [59], Reykholt in Iceland [60], Garđar and other Norse farms in Greenland [61, 62]. In contrast, the burial provides the first record of *Byturus tomentosus* (Deg.), the raspberry beetle. As its English vernacular name suggests, it may be a pest on cultivated raspberries, *Rubus idaeus* L., breeding in the drupes, but it also breeds in other *Rubus* spp. Stenseth [63] has studied the life cycle of this beetle in the Oslo region, where newly emerged adults leave pupal chambers in the soil during the first part of May when temperatures exceed 10° C, and became established on raspberry canes between one and two weeks before flowering. The adults feed on a variety of flowers and oviposition into the developing fruit takes place during the flowering season, in Norway during late June or July. Further north, emergence is likely to be delayed to later in the summer. Both Strand [64] and Lindroth [65] only record the species as far north as the southern part of inner Nordland, although it extends further north into Swedish Lapland.

## Interpretation

Interpretation of the Øksnes boat ‘burial’ presents an immediate problem: was there a body and if so, where is it? At Kvalsund at least four boats had been buried in pits in a peat bog with evident ritual but no burials [66–67]; at Øksnes the insect evidence is conclusive for the previous presence of a body. It has been assumed that peat acidity was sufficient to accelerate the process of decomposition, yet as the numerous bog bodies show [1], bogs are more likely to preserve than destroy. Whilst bone may be partially if not wholly dissolved [68], flesh and some internal organs are tanned and differentially preserved [69].

In the absence of a body, the preserved material from the boat burial reveals forensic information which is quite unique. Part of the fauna is associated with the items within the burial, e.g. the feathers as part of the pillow and perhaps its previous use. A component of the fauna is associated with the burial itself and the decomposition process, whilst several of the Coleoptera may provide information about the surrounding natural environments, although there is a problem as to whether these entered the burial prior to inhumation or were part of the peat deposits in the area where the burial took place. Although the small boat is unlikely to have had a resident inboard fauna, the beetles recovered could have been introduced in hay used as dunnage in the boat [70].

One of the species recovered, *B*. *tomentosus*, could have also entered the burial with flowers. The presence of flowers, perhaps left on the pillow, would not be out of place in a burial, for decoration, to disguise the putrid smell of decay, or as part of the burial ceremony. There is evidence of flowers in burials from the Neolithic onwards in Scandinavia [71], and pollen from a Neolithic stone cist in southern Sweden, dated to 2040–1690 cal BC shows that wood anemones, *Anemone nemorosa* L., were deposited with the body [72]. Pollen of the marsh gentian *Gentiana pneumonanthe* L. was also recovered from a Bronze Age (1500–900 cal BC) cairn at Hisingen in Göteborg, Sweden, implying deposition of flowers into the grave [73], and a single flower of yarrow, *Achillea millefolium* L. was recovered from the early Bronze Age oak coffin burial of the Egtved burial, c. 1390–1370 BC, in Vejle, Denmark [74] There also appears to have been flowers in the Oseberg ship burial [75] and both Cichoriaceae and Asteraceae flowers were found in late medieval graves at Hamina in Finland [76].

The fly fauna from the feather pillow leaves no doubt that there had been a body in the grave. As in numerous other forensic cases, *Protophormia terraenovae* would have oviposited on exposed parts of the body, perhaps the deceased's head lying on the pillow. Maggots would appear on the body several days after death and the fact that all *P*. *terraenovae* puparia found were eclosed indicates that the body had not been buried immediately after death, allowing the flies to complete their life cycle. The length of time between death and recovery of the body is difficult to estimate in that the development time of this calliphorid is dependent upon ambient temperature and moisture [77–79]. In addition, death could have equally taken place in the winter, with the body remaining frozen before blowflies took possession of the corpse during a warmer interval, prior to burial. However it is notable that there is little evidence of species associated with later stages of decay and burial [31, 80–82] posing questions about the reason behind this.

The fleas recovered from the pillow feathers provide a further problem. All were adult human fleas and there were no bird fleas or lice. The fleas may have moved into the pillow seeking warmth after the demise of the human, since fleas tend to abandon rapidly dead hosts [83], but the bilges of even a small boat, wet, saline and foul, would not have offered a suitable retreat. It is possible that the pillow came to the burial from elsewhere where the feathers had provided a nesting area for the larvae to develop [84], although there is no evidence of a breeding population, unemerged individuals or cocoon fragments, perhaps as a result of preservation. Newly emerged fleas can cope with starvation for longer periods, in particular at low temperatures [85–86], lasting in diapause without a blood meal for several weeks. The low temperatures in the grave, make it unlikely that the recovered imagines had completed their development and emerged after the burial of the pillow.

Fleas tend to abandon dead hosts [83] and although Bacot [87] observes that starving *P*. *irritans* may survive up to 135 days, in this particular case, there was no way out.

### The archaeological context

As a result of the nature of the excavation, there is limited information about the burial or specific details about the Øksnes burial. The forensic entomology, however, clearly indicates that this was a burial, rather than a cenotaph. No bones or tissue were recovered although the preservation of the feathers indicates that at least within this particular area of the bog the conditions were ideal. In terms of the insect fauna associated with death assemblages, arguing from an absence, the lack of a subterranean post-burial fauna would suggest that the corpse was removed either relatively soon after interment or the burial was at the end of the summer and the body had been removed before the ground warmed up over the following summer.

Discussion still continues about the ethnic origins of the deceased. Based on evidence for sewing, a common Sami boat building technique, Gjessing [11] and later Westerdahl [88–89] proposed a Sami origin for the Øksnes vessel. However, Gjessing’s assertion that the boat had been sewn together with reindeer sinew (idem), which he defined as a Sami trait, has been proved wrong by later analysis confirming the use of root fibres. Pedersen [90] rejects these interpretations and suggests that the boat was a Norse vessel. However, sewn boats go back at least to the Bronze Age [91], and there are both Pre-Roman Iron Age (e.g. Hjortspring in Denmark [92]) and Roman examples (e.g. Aquileia, Italy [93]). It is not possible to tie the boat construction with any ethnic group [94], the technique remaining in use into the early twentieth century around the White Sea [95].

Boat burials are commonly associated with Norse Late Iron Age traditions [96], although in this case the story might be more complex. The use of birch bark in the boat and possible wrapping of the body of the deceased in cattle hide provide some additional clues. There are medieval Sami burials where there is evidence for wrapping bodies in birch bark or reindeer hides [97, 98] and there is evidence for the use of cattle hide wrapping in Late Iron Age burials in Fennoscandia, for example, burial 40, of a female, at Kaarina Kirkkomäki [99] and a grave from Köyliö Cemetery C [100], both in Finland.

Although there has been some discussion about gender in relation to burial practices, neither the cattle hide nor other objects in the burial are associated with either male or female individuals. Axes for example have been found deposited with females, as in the Føre burial [15]. Although social status may be evident from grave offerings, there is little evidence that different items can be assigned to different genders.

The inclusion of the pillow in the boat burial might provide some additional clues. Pillows and quilts of feathers and down are known from high-rank burials dating to the Late Iron Age in Scandinavia and Western Europe [101], although these data partly reflect the limitations imposed by preservation and sites researched. The best known ship burial where a feather pillow was recovered is Oseberg, dated dendrochronologically to AD 834. Also from Norway, both the Gokstad ship burial, dated to AD 892 [7, 102] and the chamber grave at Haugen, Tune, in Østfold, dated to AD 910 [7] included feathers, perhaps from a pillow or a quilt. An older example comes from a late Vendel ship burial at Valsgärde in Sweden (grave 6, c. AD 750, Arwidsson 1942) [103]. There are various other examples (e.g. Jelling (ca. 970 AD) and Mammen (ca. 970–1080 AD) in Denmark, grave 390 at Luistari, Finland (900–950 AD), various graves at Birka, Sweden (e.g. two 9th century graves, 579 and 825 [104], and 30 later graves [105]), whilst the earliest evidence in northern Europe is a feather pillow from the 7th century Sutton Hoo ship burial [106]. The inclusion of a feather pillow at the Øksnes burial might reflect a long tradition of exploitation of feathers and down in northern Norway. The oldest reference to trade in *fugela feðerum*, bird feathers, is the account Ohthere of Hålogaland gave to King Alfred of England about AD 890 mentioning that the Sami used fugela feðerum to pay taxes [107, 101]. That feather pillows might have more significance than as a luxury item is hinted at in Erik the Red’s Saga, where the seeress Thorbjerg has a cushion on the high seat which was stuffed specifically with chicken feathers.

### An empty grave

Penecontemporaneous disturbance of burials has recently been discussed in both the Anglo-Saxon and Scandinavian context [108] and the rather simplistic hypothesis of ‘grave robbing’ has been subject to scrutiny. At both Gamla Uppsala and Vendel, Klevnäs [109, 110] considers the possibility that disturbance reflects part of the process of adoption of Christianity, although other reasons are considered. In Iceland, graves were opened and the occupants reburied in consecrated ground [111] and Christian cemeteries were cleared when farms were abandoned or relocated. Vésteinsson [112] also notes Saga and other sources for this practice. Øksnes differs, as Gjessing [11] observed, in its liminal location of a single grave mound in a bog, although he drew parallels with other boat graves. Klevnäs [109] does refer to the possibility of the exhumation and disposal of presumed revenants and a recent study of the human bones from a pit at Wharram Percy in East Yorkshire [113] provides a more graphic medieval example. Mutilated burials, where the head had been placed between the legs in Roman and Anglo-Saxon contexts, had earlier been considered by Harman et al. [114], who also raise the possibility of the walking dead, although they are more inclined to sacrificial explanations. The medieval Icelandic literature has recently been extensively reviewed by Jakobsson [115], and Caciola [116] provides a more theoretical review on a broader European scale. In the Saga literature, the case of Killer Hrapp in Laxdale Saga is perhaps the best known and most pertinent. Hrapp, a refugee from the Hebrides, had been a thug and bully during life and his wife, Vigdis, did not dare oppose his wishes in death. He insisted on burial upright at the threshold of his farm, neither inside nor out, and he continued to menace the occupants from the threshold, killing most of the servants. He also continued to cause problems for other farmers in Laxardalur, until they petitioned the local chieftain, Hoskuld, who dug up the corpse and reburied it away from the farms and their livestock, although he still haunted the region. Hrapp’s son, Sumarlidi, who had inherited the farm, went insane. Later Hrapp turns his attention to a nearby farm, Hjardarholt, where Hoskuld’s son, Olaf had taken up residence. Again standing at the threshold, he frightens off Olaf’s servant and then breaks a spear thrust at him by Olaf, keeping the spearhead. Olaf then digs up Hrapp’s body, which is perfectly preserved and has his spearhead with it. He has the corpse burnt and his ashes taken out to sea. There is no more haunting. Similar episodes of haunting, exhumation and reburial or cremation occur in several other Sagas [115]. The fight between the undead Glam and Grettir, with its echoes of Beowulf, also involves an outsider–Glam was a Swedish immigrant to Iceland. In life, potential revenants, like witches, are often marginal to the community and it is equally significant that Grettir, who has at least a little of the troll about himself, finds his eventual downfall, as Glam predicts, in his fear of the dark. It is difficult to comprehend how the dark and unknown circumscribed each isolated community, be it farm, fishery or village and one easily falls into the trap of ascribing modern neuroses to the past [117].

What is attested in the case of Øksnes, is the burial of a body, perhaps shipwreck victim ashore (see S1 File) or of an outsider marginal to the community, followed shortly afterwards by its exhumation.

## Conclusions

Although limited preservation as a result of human impact and peat cutting in the area has set limitations for the archaeological study of the Øksnes assemblage, several decades after the excavation of the boat the study of the insect remains from the feathers recovered from the site provided new data about aspects of the burial. With results which range from the surrounding environment to intimate information about the particular context, fossil insects provide some interesting details:

- Although there is no deceased, *Protophormia terraenovae* indicates that there had been a burial, associated with the feather pillow, and that the corpse had been exposed for several days before burial.
- The beetle and the fly information point to a burial during a period of warm weather, perhaps the end of spring or summer.
- The evidence from *Pulex irritans*, the human fleas, found within the feathers, indicates that the pillow deposited in the boat had probably come from a domestic context.
- The beetle fauna recovered was typical of hay assemblages, perhaps from the bilges of the boat, whilst species such as *Byturus tomentosus* might suggest that flowers had been deposited with the burial.
- The lack of a body coupled with the lack of post burial fauna would support the hypothesis that the corpse had been removed and disposed of elsewhere; historical and Saga sources point to similar examples of exhumations because of perceived revenant activities.
- Past burial insect assemblages are a powerful tool for the understanding of these forensic scenes. Fossil insect and paleoecological research have the potential to significantly enhance our understanding of grave assemblages adding much needed detail both about the particular sites and specific data on the environment and the social context of the burial, even in the absence of an actual body.

## Supporting information

S1 File A possible scenario. (DOCX)
