# Supplementary material for: Is there anybody in there? Entomological evidence from a boat burial at Øksnes in Vesterålen, northern Norway
Source: PLoS One. 2018 Jul 27;13(7):e0200545. doi: 10.1371/journal.pone.0200545 (PMC6063414; doi:10.1371/journal.pone.0200545)
Supplement: S1 File — (DOCX) [file pone.0200545.s001.docx]

**S1 File: A Possible Scenario**

A boat drifts ashore near Øksnes containing a single corpse with some damage from feeding maggots but otherwise substantially intact. The local community involved in fisheries are probably used to recovering the drowned and those dead from exhaustion or hypothermia, and burying them, but this one is different, either in garb or nature of the boat, perhaps still sewn when most local ones were clinker built with iron rivets. Where had the individual come from, or was it, like Peter Grimes in Britten’s opera, someone known but always marginalised in the community by manner, religion, ethnic or social background? The community decide that a burial is in order, a boat burial utilising the wreck and including the person’s belongings, principally an axe. The boat with its occupant is buried at the edge of the bog, perhaps with a pillow hiding the face. The location is liminal, neither on land nor in water, but even with its ring of stones separating the living space from the dead, it fails to stop the dead from walking. The body has to be removed and disposed of; there are no more perceived hauntings.
